# On the Importance of Contrasts in Taxonomic Diagnoses: A Survey of 405 Newly Described Insect Genera

**DOI:** 10.3390/insects16121224

**Published:** 2025-12-01

**Authors:** Laurence Packer

**Affiliations:** Department of Biology, York University, 4700 Keele St., Toronto, ON M3J 1P3, Canada; xeromelissa@gmail.com

**Keywords:** taxonomic practice, biodiversity assessment, ease-of-use, best practices, international code of zoological nomenclature

## Abstract

Diagnoses are one of the most important ways that taxonomists make new taxa recognisable by others. A recent paper stated that diagnoses for newly described organisms should include both the characteristic(s) of the new taxon (providing state-specificity) and name the taxa with which they are compared (giving contrastiveness). I argue that the characteristics of the compared taxa should also be included such that the diagnostic features are not only contrastIVE but also overtly contrastED I surveyed 278 papers wherein 405 new insect genera were described. Forty-four genera did not have a formal diagnosis, among the rest, there was a total of 427 diagnoses because some genera had multiple diagnoses. Among these, 83 (19.4%) had one or more diagnostic states overtly contrasted and over one-eighth (13.9%) contrasted all of them. Unsurprisingly, diagnoses that overtly compared the new genus to others were significantly more likely to include characteristics that were contrasted than did diagnoses that were lists or combinations. I discuss how features should be contrasted in diagnoses.

## Simple Summary

Descriptions of new taxa are usually accompanied by a diagnosis that explains how to differentiate it from others. This differentiation is made easier when the features of the new taxon are overtly contrasted to those with which it is being compared. I find that most diagnoses do not provide appropriate contrasts to facilitate differentiation.

## 1. Introduction

While many taxonomists believe diagnoses are a requirement of new taxon descriptions, their inclusion is merely a recommendation according to the International Commission on Zoological Nomenclature (see Recommendation 13A, [1] p. 17). Additionally, there is little agreement on how diagnoses should be framed. Rheindt et al. [2] argue that the “gold standard” for a diagnosis is that it contains one or more features that are both state specific and contrastive. An example they give is leg colour for which they provide “green legs” as an example of state specificity and three possible examples of a contrastive diagnosis (Box 2, 4th page): (i) “New Species X differs in leg colour from Species Y”; (ii) “The green leg colour of new Species X is unique among members of its genus” and (iii) “New Species X has green legs, while Species Y has red legs”. Only the last of those three examples tells the reader what the alternative condition is. A user confronted with either of the other two options may be confused as to whether or not a specimen with yellow-green, brown-green or turquoise legs is New Species X. I argue that in addition to being state specific and contrastIVE, an optimal diagnosis would also be contrastED.

In support of the view that diagnoses should include the contrasted states overtly, I present the results of assessments of 427 diagnoses from 278 papers associated with the description of 361 new insect genera and investigate the extent to which diagnostic features were contrasted. I find that many authors include contrasted feature states in their diagnoses but that the proportion depends upon the style of the diagnosis. I conclude that taxonomy would be improved if all diagnoses clearly stated the contrasted features of the other organisms in the group to which the new taxon belongs.

## 2. Materials and Methods

Papers on newly described extant insect genera were obtained based on Scopus searches for insect ordinal names and “new gen*” and synonymous terms as outlined in detail elsewhere [3]. Papers found in those searches published over an 18-month period starting in January 2021 which included one or more diagnoses for new extant genera were included in the dataset treated herein.

I define the group with which the new genus may be contrasted as the reference group [4]. For example, if the diagnosis contrasts a newly discovered genus only with the genus it would have previously keyed to, then the reference group is the latter genus. Alternatively, if the diagnosis of the new genus contrasts it with all other genera in a tribe, then the reference group is that tribe.

I found that all diagnoses could be placed into one of three categories: lists, combinations and comparisons [4]. Lists were unqualified statements where feature states were given in paragraph or itemised form. Combinations were like lists except that it seemed clear that a specimen had to agree with all feature states provided. These almost always used a word based upon “combin” (“combination” or “combined”) but occasionally the word “complex” was used, and I took this to mean all subsequent features had to agree with the diagnostic statements (e.g., [5] “the male genitalia of ….. are characterised by the complex of the following characters:”). Comparison diagnoses compared the new genus with those found in the reference group. These generally involved statements that a subset of taxa shared a state for one or more features with the new genus but differed in some others and often used different suites of features for different subsets of taxa within the reference group. While it was usually easy to determine which of the three categories a diagnosis belonged to, it was sometimes difficult, usually as a result of different categories being mixed within the same paragraph.

As an empirical example of the utility of the three different categories of diagnosis, as well as the term reference group, consider [6]. These authors included three different diagnosis types each associated with a different reference group for their new genus *Siccasura* (Lepidoptera: Erebidae): a list contrasted it with a species group within another genus, a combination of male genitalic states separated it from one genus and a series of comparisons of male and female genitalic features was made to all other genera in a generic complex.

When multiple diagnosis types were detected, they were treated separately, resulting in the number of diagnoses in the dataset being larger than the number of new genera. While comparisons to the most similar relatives were commonly provided after a list or combination, they were treated separately as the reference groups implied were different. Nonetheless, only three genera in the sample received more than one type of diagnosis which included contrasts.

As with analyses of identification keys [3], I use the terms feature and feature state instead of character and character state, e.g., [2,7], to avoid implication that the observations under discussion might be part of a phylogenetic analysis. Though it is worth noting that surprisingly few of the new genera were associated with any phylogenetic treatment and so the justifiability of many of them remains undemonstrated [8,9].

I checked each diagnosis for overtly contrasted features. A good example would be “X [the new taxon] has an acute spine apicodorsally on the hind tibia whereas in genus Y this area is smoothly rounded”. A diagnosis that was not contrasted (but remained contrastive and state-specific) might say “X has an apicodorsal spine, a condition not found in Y”. The latter is clearly less decisive because perhaps species Y has an acute process which may be confused with a spine: it is preferrable to state alternatives in the positive. Many comparative statements were not so clearly differentiated. For example, if the text stated that structure Z was wider in the new taxon and narrower in the reference group (i.e., the contrasted taxa) this was not considered as an overt contrast unless details of relative length to width of the feature were also provided. Less usefully, diagnoses sometimes included qualifiers such as “usually”, “generally”, etc. These were not considered to be contrasted because (a) potential users would not be able to use such features to identify a specimen possessing the unusual condition and (b) this cannot be evaluated from a single specimen, which is the way users of taxonomic information will normally proceed.

For each diagnosis I counted the number of feature states that were provided to differentiate the new genus from others and how many were overtly contrasted. Because of the complexity of the information provided in many diagnoses and their frequent lack of clear structure, this was often difficult, particularly for comparison diagnoses where differences to different subsets of the reference group often had to be assessed or when different diagnosis types (lists, combinations, comparisons) were intermingled within a paragraph. Additionally, it was frequently not clear how many differences were implied in the description of a single complex structure. To give an entirely concocted example, consider the following: bromus narrow, elongate, apex turned upwards and concave, with a pair of swellings at the base. Is this one feature, five or some number in between? The logical way to answer the question would be to consider the number of independently evolving features. In the absence of a formal phylogenetic analysis, this is something that even authors describing new genera might not know. For the above reasons, my estimates may have been made with some error in comparison to the number of features the authors might have had in mind.

Only differences, and not similarities, were included in enumeration as the latter were unlikely to be contrasted and may not need to be. Additionally, similarities were often not given (e.g., for *Majialandrevus*, Orthoptera: Gryllidae, [10] were vague (e.g., “superficially similar to” for *Scarlata*, Lepidoptera: Sesiidae, [11] or even “vaguely reminiscent of” for *Burmanyctycia*, Lepidoptera: Noctuidae, [12]) or based entirely on molecular data (e.g., for *Cisandina*, Lepidoptera: Geometridae, [13]).

All analyses were performed on a per-diagnosis basis because that is the way they are most likely to be used. This probably did not result in any statistical bias towards the approaches adopted by authors of papers describing larger numbers of genera because the maximum number of contrasted diagnoses in a paper was two, even if a larger number of new genera were described (e.g., [14] describes 11 new genera, but only one diagnosis included overt contrasts).

## 3. Results

The literature sample comprised descriptions of 405 new genera in 278 papers. However, no sections with subtitles including words such as “diagnosis” or “diagnostic” were found for 44 genera described in 36 papers. Thus, there were diagnoses for 361 genera in a total of 243 papers (one paper had one genus described with a diagnosis and two without). The total number of diagnoses available for assessment was 427, which is greater than the number of new genera because of separate diagnoses for males and females and/or more than one type of diagnosis being provided for a genus, usually with different reference groups. Full tabulation of the results for all diagnoses with contrasts is provided in Table 1.

The largest category of diagnosis was the list, which was used 225 times (52.7% of the total number of diagnoses), comparisons came second (129 instances, 30.2%) and combinations third (73 cases, 17.1%). A total of 83 diagnoses (19.4% of the total) had one or more feature states overtly contrasted, but this varied among diagnosis types. Of the 225 list diagnoses, only five (2.2%) had any feature states contrasted. Four (5.5%) of the combination diagnoses included one or more contrasts. Unsurprisingly, comparison diagnoses included contrasts significantly more often (in 74 cases—57.4%; χ^2^ = 179.2, *p* < 0.00001).

Over all diagnoses that included at least one contrast, most feature states were contrasted (on average 76.4% were), and 50 (60.2%) contrasted all of them. Because of the greater increase in effort required, it might be expected that diagnoses with a more extensive suite of features provided might have a smaller proportion of states overtly contrasted. However, the relationship between number of features and the proportion that were contrasted was not significant (r_s_ = −0.099, *p* = 0.38) and there was no difference in the number of feature states given in diagnoses where all features were contrasted in comparison to those in which only some were (mean # features listed for the new genus where all were contrasted = 6.32, mean # when not all were contrasted = 7.48; z = −1.23, *p* = 0.22).

While the number of combination diagnoses with contrasts was too small to include in statistical analyses, there were no significant differences in number of feature states or the percentage of them that were contrasted between list and comparison diagnoses (Kruskal–Wallace test, H = 0.054, *p* > 0.8 and H = 1.03, *p* > 0.3, respectively).

## 4. Discussion

Diagnoses are one of the most important aspects of new taxon descriptions [79,80] and in the absence of an identification key are likely the main means whereby the new taxa can be identified by those other than the authors [3,81]. While there are no generally accepted ways in which diagnoses should be framed, Rheindt et al. [2] suggest that the “gold standard” involves not only stating the observation for the new taxon (providing state-specificity) but also naming the taxa which differ (providing contrastiveness). I argue that to be of greater utility, a diagnosis should also clearly state the alternative potential observations found in the reference group (those organisms to which the new taxon is contrasted in the diagnosis). In the three hypothetical examples given in [2]—Box 2, only one stated the alternative state (see Introduction). It seems that some taxonomists understand the importance of providing contrasted feature states, as at least one was given in almost one-fifth of new genus diagnoses in my sample, and over 60% of those contrasted all of them. These numbers are likely somewhat underestimated given that my criteria for including a feature state as having been contrasted were rather stringent. For example, stating that a structure is present implies that the alternative state would be absent, but unless that was overtly mentioned I did not consider it sufficiently contrasted (for example, an alternative to a spine might be an angulation rather than a flat area, see Methods). Similarly, all combination diagnoses might be considered as contrasted because other taxa would lack one or more of the feature states within the combination. If all combination diagnoses are taken to be contrasted, then the total number of diagnoses that include contrasts increases to 152 (35.6% of all diagnoses). However, each feature state in a combination diagnosis must be checked to confirm an identification and each of these observations are easier to understand if they are properly contrasted. Thus, it seems better to provide overt contrasts for each feature state whether the diagnosis is a combination or not.

It might be expected that all comparison diagnoses should include contrasts, but this was not the case because I did not include statements that were too vague for easy understanding. For example, the statement “compared to genus X, Y is larger, has narrower wings and the male genitalia has a large spine on the penis valve” has three feature states compared but none appropriately contrasted. Appropriate contrasts in this hypothetical example would provide measurements for body size for the focal taxon and its reference group, length to width ratios for both wider and narrower wings and state what the reference group had on the penis valve in the location where the new taxon had a spine.

Some discussion of what makes an appropriate contrast is required. As noted in the Methods, relative statements are generally not very useful. For example, stating that a colour band on the forewing is narrower in the new taxon compared to others is not useful unless either (i) contrasted relative measurements are given (e.g., length to width at least 4:1 versus at most 3:1) or (ii) both are imaged—and, given intrataxon variation, choice of images here is crucial “at least as wide as in figure K versus at most as wide as in figure L,” (where K shows the narrowest example of a wide band and L the widest example of a narrow band). Additionally, alternative states should be given in the positive; rather than saying that a spine is absent, describe the shape around where the spine would have been if it were present: again, would an acute angulation count as being a spine?

Deciding upon the exact wording for contrasting states may often be difficult. For example, if green legs are diagnostic and a more detailed contrast is desired than simply stating “legs not green”, what range of colours are found in the alternatives and how close to “green” might a colour be without counting as being green? Who among us have never had a disagreement with someone about whether a particular object is turquoise or green, or blue (see [82])? Similarly, if the complex shape of a genitalic feature is diagnostic, the range of alternatives is potentially almost infinite and listing and/or imaging all of them impractical. In such cases stating what the most similar alternatives are and illustrating them with the figures close to those of the new taxon to facilitate comparison would seem a sensible solution. The text outlining the contrasted feature state can be emended to “at least as different from figure A (which is of the taxon being diagnosed) as shown in figures B or C.”

Rheindt et al. [2] concentrated on species-level diagnoses and was largely oriented towards DNA-based identifications. Nonetheless, their statements about what taxonomists consider to be appropriate diagnoses were not restricted to molecular data; morphological examples were given, and their arguments should certainly apply to all levels in the taxonomic hierarchy, at least with respect to morphological data. They also suggest that the upcoming fifth edition of the zoological Code should stipulate that a diagnosis should “clearly provide(s) information on at least one specific character and its character state in which the new species differs from closely related species.” I suggest that the Code should also stipulate that the alternate state(s) in the taxa for which the diagnosis is contrastive (i.e., the reference group) should also be provided. This requirement should not be problematic for taxonomists as many already demonstrate their understanding of the importance of describing alternative, contrasted feature states by including them in their diagnoses. Furthermore, most that include such contrasts provide them for all feature states. The authors [2] further welcomed additional discussion on the topic of contrastiveness and state-specificity, something that has some urgency given the impending revision of codes of nomenclature. I hope that this paper is seen as a contribution to this discussion.

## 5. Conclusions

Taxonomic diagnoses explain how to differentiate a taxon from others by providing a suite of potential observations against which a specimen can be checked. A minority of authors state the potential alternative observations found in other taxa. To make it easier for users of taxonomic research, it should be mandatory for a diagnosis to state what these alternative observations might be for the taxa against which the new one is being contrasted. 

## Figures and Tables

**Table 1:** Data extracted from surveyed papers sorted alphabetically by author.

				Reference	# Contrasts
Paper ^a^	Genus	Order	Family	Group ^d^	/# Features
[15]	*Caribesialis*	Megaloptera	Sialidae	family	4/4
[16]	*Apolemotermes*	Blattodea	Termitidae	subfamily *	7/7
[16]	*Koutabatermes*	Blattodea	Termitidae	subfamily *	4/4
[17]	*Australara*	Coleoptera	Elmidae	subfamily *	7/7
[18]	*Soqotranus*	Coleoptera	Ptinidae	family	7/7
[12]	*Burmanyctycia*	Lepidoptera	Noctuidae	genus grp	11/11
[19]	*Salaziella*	Hemiptera	Derbidae	tribe	2/4
**[20]**	*Aurisetiphora*	Diptera	Phoridae	subfamily	2/4
[21]	*Photinopygus*	Coleoptera	Staphylinidae	subtribe	12/12
[22]	*Spinopholidoptera*	Orthoptera	Tettigoniidae	tribe	16/16
[23]	*Bozidaria*	Coleoptera	Leiodidae	genus grp	15/15
**[24]**	*Valallylum*	Orthoptera	Tetrigidae	tribe	7/22
[24]	*Valallylum*	Orthoptera	Tetrigidae	tribe	7/7
[25]	*Ruschiola*	Diptera	Cecidomyiidae	tribe *	2/2
[26]	*Ptorthus*	Hemiptera	Kinnaridae	genus grp ?	1/5
[27]	*Ebaiotrigona* ^e^	Hymenoptera	Apidae	tribe *	12/12
[28]	*Parkerodynerus*	Hymenoptera	Vespidae	genus grp	1/5
[28]	*Bohartodynerus*	Hymenoptera	Vespidae	genus grp	7/7
[29]	*Ysachron*	Coleoptera	Cerambycidae	tribe	5/5
[30]	*Arumatia*	Phasmatodea	Diapheromeridae	subfamily	2/12
**[14]**	*Papuacryptus*	Coleoptera	Cryptophagidae	tribe	1/4
[31]	*Balleriodes*	Coleoptera	Hybosoridae	subfamily	1/4
*[32]*	*Ebenacobius*	Coleoptera	Curculionidae	tribe *	1/5
[33]	*Makalapobius*	Orthoptera	Trigonidiidae	genus grp	11/11
[33]	*Gabusibius*	Orthoptera	Trigonidiidae	genus grp	11/11
[34]	*Parahabetia*	Orthoptera	Tettigoniidae	tribe	9/17
[35]	*Nyxia*	Hymenoptera	Ichneumonidae	subfamily *	2/5
[36]	*Tsounkranaglenea*	Coleoptera	Cerambycidae	tribe	2/6
[10]	*Majialandrevus*	Orthoptera	Gryllidae	genus grp ?	1/2
[37]	*Platydrilus*	Coleoptera	Telegeusidae	family	8/8
[37]	*Stenodrilus*	Coleoptera	Telegeusidae	family	8/8
[38]	*Concavetettix*	Orthoptera	Tetrigidae	genus grp	7/7
[39]	*Lafontaineana*	Lepidoptera	Noctuidae	one genus	6/8
[13]	*Knudsonia*	Lepidoptera	Geometridae	tribe	2/4
[40]	*Prokius*	Hymenoptera	Cynipidae	tribe	3/4
[41]	*Telmatometropsis*	Hemiptera	Gerridae	subfamily *	7/8
[42]	*Cisandina*	Lepidoptera	Nymphalidae	genus grp	6/6
[43]	*Mahabadphora*	Diptera	Phoridae	subfamily	1/4
[44]	*Vietnalia*	Coleoptera	Tenebrionidae	subfamily	7/7
[45]	*Dubianus*	Dermaptera	Forficulidae	order *	2/2
[46]	*Urbanthidium*	Hymenoptera	Megachilidae ^c^	subtribe	3/5
[47]	*Viperinus*	Lepidoptera	Lecithoceridae	one genus	1/3
[48]	*Spiniola*	Lepidoptera	Lecithoceridae	genus grp	2/2
[49]	*Vietanna*	Hemiptera	Cicadidae	subtribe	6/6
[50]	*Rudogorites*	Coleoptera	Leiodidae	genus grp	1/7
[51]	*Spanglerelmis*	Coleoptera	Elmidae	family	6/6
[52]	*Sahyadrialtica*	Coleoptera	Chrysomelidae	genus grp	4/4
[53]	*Carinadelius*	Hymenoptera	Braconidae	tribe	8/8
[54]	*Graziasternum*	Hemiptera	Pentatomidae	1 genus	7/7
[55]	*Nelbroma*	Hemiptera	Cicadidae	tribe	4/4
[56]	*Ebogotermes*	Blattodea ^b^	Termitidae	subfamily *	2/2
[57]	*Compsogusa*	Mantodea	Gonypetidae	one genus	2/4
*[58]*	*Protrachyasmus*	Coleoptera	Curculionidae	one genus	14/14
[59]	*Michener*	Hymenoptera	Braconidae	subfamily	4/4
**[60]**	*Miguelmonneus*	Coleoptera	Cerambycidae	genus grp ?	1/8
[11]	*Scarlata*	Lepidoptera	Sesiidae	genus grp	2/6
[11]	*Malayomelitta*	Lepidoptera	Sesiidae	genus grp	3/8
[61]	*Acanthamoplax*	Hemiptera	Tingidae	family	7/7
[62]	*Selizitapia*	Hemiptera	Flatidae	tribe *	5/5
[63]	*Makaya*	Hemiptera	Flatidae	tribe	5/5
[64]	*Birabiro*	Lepidoptera	Lycaenidae	genus grp	1/3
[65]	*Rugabinthus*	Orthoptera	Gryllidae	subtribe	4/8
[66]	*Fadinthus*	Orthoptera	Gryllidae	subtribe	2/9
[66]	*Falcerminthus*	Orthoptera	Gryllidae	subtribe	3/10
[67]	*Warimiri*	Orthoptera	Tettigoniidae	genus grp ?	3/3
[68]	*Helioandesia*	Lepidoptera	Heliodinidae	family	6/6
[69]	*Zaragozapirhidius*	Coleoptera	Ripiphoridae	subfamily	3/3
[70]	*Sinatablatta*	Blattodea	Blatellidae	one genus	1/1
[70]	*Antroxestoblatta*	Blattodea	Blatellidae	genus grp	2/2
[71]	*Ratsa*	Lepidoptera	Geometridae	two genera	3/11
[72]	*Platycesta*	Coleoptera	Chrysomelidae	genus grp	3/3
[73]	*Pyesexora*	Coleoptera	Chrysomelidae	tribe	1/1
[73]	*Yingaburxia*	Coleoptera	Chrysomelidae	<tribe	1/1
[74]	*Juxtilema*	Lepidoptera	Erebidae	subfamily	17/18
*[5]*	*Setteleia*	Lepidoptera	Erebidae	genus grp	12/12
[5]	*Setteleia*	Lepidoptera	Erebidae	two genera	2/2
**[6]**	*Siccasura*	Lepidoptera	Erebidae	species grp	3/3
*[6]*	*Siccasura*	Lepidoptera	Erebidae	genus	3/3
[6]	*Siccasura*	Lepidoptera	Erebidae	genus grp	6/6
[75]	*Kruegerilema*	Lepidoptera	Erebidae	one genus	8/8
[76]	*Sabzia*	Lepidoptera	Geometridae	two genera	12/13
[77]	*Halopanurgus*	Hymenoptera	Andrenidae	genus grp	8/8
[78]	*Asicimbex*	Hymenoptera	Cimbicidae	subfamily	15/15

^a^ all diagnoses are comparisons unless given in bold—lists, or underlined italics—combinations. ^b^ treated as Isoptera by authors. ^c^ treated as Apidae by authors. ^d^ * indicates the reference group was a geographical, ecological or morphological subset, ? Indicates uncertainty ^e^ workers only.

## Data Availability

The original contributions presented in this study are included in the article. Further inquiries can be directed to the corresponding author.
